# Enduring maternal brain changes and their role in mediating motherhood’s impact on well-being

**DOI:** 10.1038/s41598-024-67316-y

**Published:** 2024-07-18

**Authors:** Valentina Rotondi, Michele Allegra, Ridhi Kashyap, Nicola Barban, Maria Sironi, Carlo Reverberi

**Affiliations:** 1 SUPSI, Lugano, Switzerland; 2 University of Oxford & Nuffield College, Oxford, UK; 3 Milan Center for Neuroscience - NeuroMI, University of Milano-Bicocca, Milan, Italy; 4 University College London, London, UK; 5 University of Bologna, Bologna, Italy; 6 University of Padua, Padua, Italy; 7 Department of Psychology, University of Milano - Bicocca, Milan, Italy

**Keywords:** Neuroscience, Social evolution, Psychology, Human behaviour

## Abstract

Parenthood, particularly motherhood, is known to impact the structure and function of the brain in the short term, but the long-term effects of parenthood and their impacts on well-being are still poorly understood. This study explores the potential longer-term associations between parenthood and the brain, parenthood and well-being, and the potential role of brain modifications in influencing mothers’ well-being. Using data from the UK Biobank, which includes brain imaging information from individuals aged 45–82 at the MRI scanning, we discovered differences in brain structure between mothers and non-mothers, with mothers exhibiting widely distributed higher gray matter density, particularly strong in frontal and occipital regions. No brain changes were observed in fathers. Parents reported a higher sense of life’s meaning compared to their childless counterparts. Gray matter changes did not mediate the relationship between motherhood and well-being. This suggests that the alterations in gray matter associated with motherhood do not play a deterministic role in shaping long-term changes in well-being.

## Introduction

Parenthood universally stands as a profound transformation, reshaping individuals’ lives and identities in myriad ways. For many, this journey radically alters life perspectives and priorities, infusing a deep sense of joy and fulfillment through the act of raising children. Yet, it also ushers in its share of challenges, requiring adjustments to new routines and increased responsibilities. This evolution encompasses the minutiae of daily life and momentous, life-defining experiences.

Neuroscientific investigations have shown that parenthood brings significant transformations not only in the lives and behaviors of mothers but also within their brains, especially throughout pregnancy and the postpartum period^1–12^. A longitudinal study by^3^ revealed a pre-post pregnancy *decrease* in gray matter density, mainly affecting the Theory of Mind (ToM) brain network. The ToM regions are associated with the ability to infer both cognitive and affective states of others and utilize this information to predict their behavior^13,14^. In contrast to the reductions observed during pregnancy, later studies have identified *increases* in gray matter density in the postpartum period^8,15^, indicating a potential recovery or enhancement of gray matter volume in mothers. Additionally, cross-sectional research has highlighted a deceleration in the typical age-related loss of gray matter among mothers^5,12,16^. For an overview of this body of work, see^15,17,18^. Also, fathers’ brains may be sensitive to caring for children, although the empirical evidence for these changes is more mixed than for mothers^19–22^.

Despite these insights, our comprehension of the long-term neural impact of parenthood remains incomplete.

Most longitudinal studies cover up to two years post-birth, except for^23^’s research, which observed brain changes in seven mothers over six years. This scarcity of long-term data leaves significant gaps in our understanding of motherhood’s enduring neurological effects. Moreover, while larger cross-sectional studies have proposed that mothers’ brains may exhibit a “younger” phenotype relative to those of non-mothers, the specific nature of the gray matter alterations underlying this phenomenon is yet to be fully elucidated. This uncertainty prompts several pivotal questions: Does the pre-post pregnancy decrease in gray matter volume within the ToM regions, as reported by^3^, reverse over time? Might there be a restoration or even an increase in gray matter volume in other areas, as suggested by^8^? Or does pregnancy confer a broad, nonspecific protective effect on the brain, influencing both cortical and subcortical structures? Additionally, it is crucial to ascertain whether the brain changes observed are unique to mothers, pointing to a biological underpinning, or if similar alterations occur in fathers, suggesting an influence of the parenting role itself. Resolving these questions is essential to unravel pregnancy’s complex, long-term impacts on the maternal brain.

From a social science perspective, parenthood is often portrayed as both a demanding and enriching role, significantly influencing parents’ well-being^24^. While prevailing views suggest that the responsibilities and rewards of child-rearing can enhance parental satisfaction and happiness, empirical research on the link between parenthood and well-being offers mixed results. Some studies report a positive correlation, emphasizing the cognitive and affective dimensions of subjective well-being e.g.,^25^ while others present inconsistent or negative findings e.g.,^26–29^.

This study examines the long-term relationship between parenthood, the human brain, and the parents’ well-being, testing a potential connection between these dimensions.

First, we determine whether the specific brain changes observed postpartum (i.e., within six months after birth) in mothers’ brains^3^ are still detectable decades after childbearing. Second, we explore whether child-rearing experience produces similar long-term consequences in fathers’ brains. Third, we test whether gray matter density in these brain regions mediates the association between motherhood and subjective well-being in its multiple components. To answer these questions, we rely on the *UK Biobank Dataset*, which includes magnetic resonance imaging (MRI) data from participants aged 45–82 at the MRI scanning. With a sample size exceeding 19,000, this dataset offers a substantial advantage over previous studies examining brain changes in response to pregnancy that rely on primary data, where the sample size is typically less than 100, although it lacks a longitudinal setup.

In our third line of inquiry, we delve into the relationship between brain changes, particularly in areas pivotal for empathy and understanding others, and their potential impact on the mothers’ well-being. This exploration is grounded in a wealth of literature suggesting that evolutionary adaptations have refined maternal instincts to respond acutely to their children’s emotional states in species where offspring depend heavily on parental care. This evolutionary advantage, well-documented in primate studies, underscores the importance of the Theory of Mind (ToM) and empathy in effective parenting. Such capabilities empower parents, especially mothers, to accurately interpret and respond to their children’s mental and emotional cues^30–32^. Research indicates that motherhood significantly amplifies these mentalizing abilities, resulting in distinctive neural patterns when mothers process their children’s emotional signals compared to those of adults, thereby enhancing their capacity to empathize with others’ emotional states^33,34^. This double-edged sword of heightened empathy can enhance or exacerbate emotional distress through shared joy or pain. The literature highlights the latter, suggesting a predisposition towards being more affected by distress^35,36^. This suggests that the changes in the brain that occur during motherhood may impact well-being in a way potentially difficult to detect.

Our analysis proceeds in multiple steps. First, we aim to describe the long-term changes in parents’ brains. Initially, we focus on a-priori brain regions previously associated with motherhood-related gray matter changes^3^. This targeted approach enhances the analysis’s statistical robustness and ensures parameter estimates’ reliability. We separately analyze male and female participants, controlling for the effect of age. Second, we investigate whether additional brain regions exhibit a parenthood effect, and, in this case, we assess whether such effects are anatomically specific or generalized. To achieve this, we repeat the previous a-priori analysis across 400 brain parcels^37^ encompassing the entire brain. Third, we contextualize our findings regarding brain adaptations within the broader discourse on parenthood and its possible consequences in terms of well-being. Using a Probit model, we estimate the probability of experiencing well-being, as measured by feelings of happiness, meaning in life, fed-upness, and emotional distress, conditional on parenthood status (by sex) and a set of controls. This approach allows us to assess the direct association between parenthood and well-being. We then employ a mediation analysis to examine the relationship between parenthood and well-being, considering the intermediary role of brain changes. Specifically, we seek to quantify the direct association between parenthood and well-being, separate from the indirect associations mediated by alterations in the brain. This distinction is crucial for understanding not only *if* but also *how* parenthood influences a parent’s well-being. Are the observed changes in well-being a result of the brain’s adaptation to parenthood, or are they a more general consequence of the parental role? Ultimately, this investigation serves as a bridge, linking the biological and social aspects of parenthood to well-being.

## Results

### Parenthood and the brain

**Figure 1:**
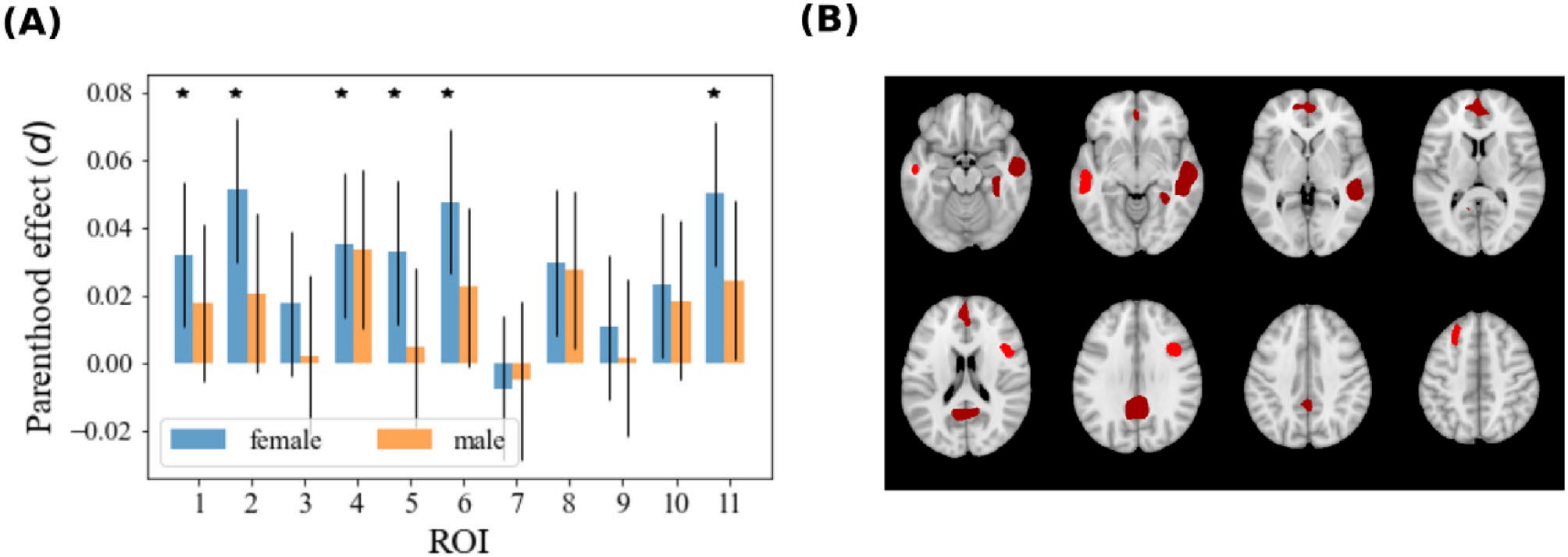
We considered 11 regions-of-interest (ROI) from^3^, and we performed a ROI-wise linear regression on grey matter density. For each region, we tested the significance of the regressor associated with parenthood. (**A**) We show the effect (Cohen’s *d*) in each region for female and male subjects. Error bars represent 95% confidence intervals. Significant ROIs ($p<0.05$, Bonferroni-corrected for 11 comparisons) are marked with an asterisk, * (**B**) ROIs presenting significant effects in women.

Comparing a sample of women before and after pregnancy,^3^ observed gray matter volume reduction in a group of brain regions. These regions partially overlap with the so-called theory-of-mind network, a network involved in social cognition. We hypothesized that parenthood could be associated with persistent differences in gray matter within these regions. Consequently, we assessed the relationship between parenthood and gray matter density in the Regions-Of-Interest (ROIs henceforth) previously identified by^3^. Since aging entails strong grey matter reductions, controlling for age is particularly important. We analyzed two age-matched subsamples, one comprising parents and the other non-parents, to minimize potential age-related biases. Within each ROI, we examined the possible effects of parenthood using multivariate linear regression, including several nuisance covariates (see Methods for details). We separately analyzed female and male participants. Results are summarized in Fig. 1 and Table 1. All linear models were globally significant: for all regions, and both males and females, we have $p < 10^{-14}$ (F-test, corrected for 11 multiple comparisons). For female participants, we found significant and *positive* effects of parenthood on grey matter density in 6 out of 11 ROIs (t-test, $p<0.05$ corrected for 11 multiple comparisons). This means that motherhood mitigates the effects of physiological neuronal loss associated with aging, although the specific causal mechanism remains unknown, potentially involving reduced neuronal loss or neuronal growth. We also found a significant effect of parenthood when considering all ROIs as a single region (Cohen’s $d=0.042$, $p=0.0001$). By contrast, we did not observe any significant parenthood effect for males. This negative result is also confirmed when all ROIs are considered globally as a single region ($d=0.021$, $p=0.09$). This lack of significant effects in males underscores the potential sex-specific nuances in the relationship between parenthood and brain structure.

**Table 1:** Parenthood-related gray matter increases in ROIs from Hoekzema et al.

ROI	Females		Males	
	Cohen’s d	*p*-value	Cohen’s d	*p*-value
1: Superior temporal sulcus, middle and superior temporal gyrus, parahippocampal gyrus R	0.032	0.039	0.018	1.00
2: Inferior frontal gyrus R	0.051	$3\times10^{-5}$	0.021	0.9
3: Fusiform gyrus, inferior temporal gyrus R	0.017	1.00	0.003	1.00
4: Superior medial frontal cortex, anterior cingulate cortex, medial orbitofrontal cortex L/R	0.035	0.02	0.034	0.06
5: Precuneus, posterior cingulate cortex L/R	0.033	0.03	0.004	1.00
6: Middle and superior frontal gyrus L	0.048	$1 \times 10^{-4}$	0.022	0.7
7: Hippocampus, parahippocampal gyrus L	− 0.007	1.000	− 0.005	1.00
8: Inferior orbitofrontal gyrus, inferior frontal gyrus, insula	0.030	0.07	0.027	0.24
9: Fusiform gyrus, inferior temporal gyrus L	0.010	1.000	0.001	1.00
10: Inferior frontal gyrus R	0.023	0.40	0.018	1.00
11: Superior temporal sulcus, middle and superior temporal gyrus, parahippocampal gyrus L	0.050	$5\times10^{-5}$	0.024	0.46

* P*-values were corrected for 11 multiple comparisons with Bonferroni correction

**Figure 2:**
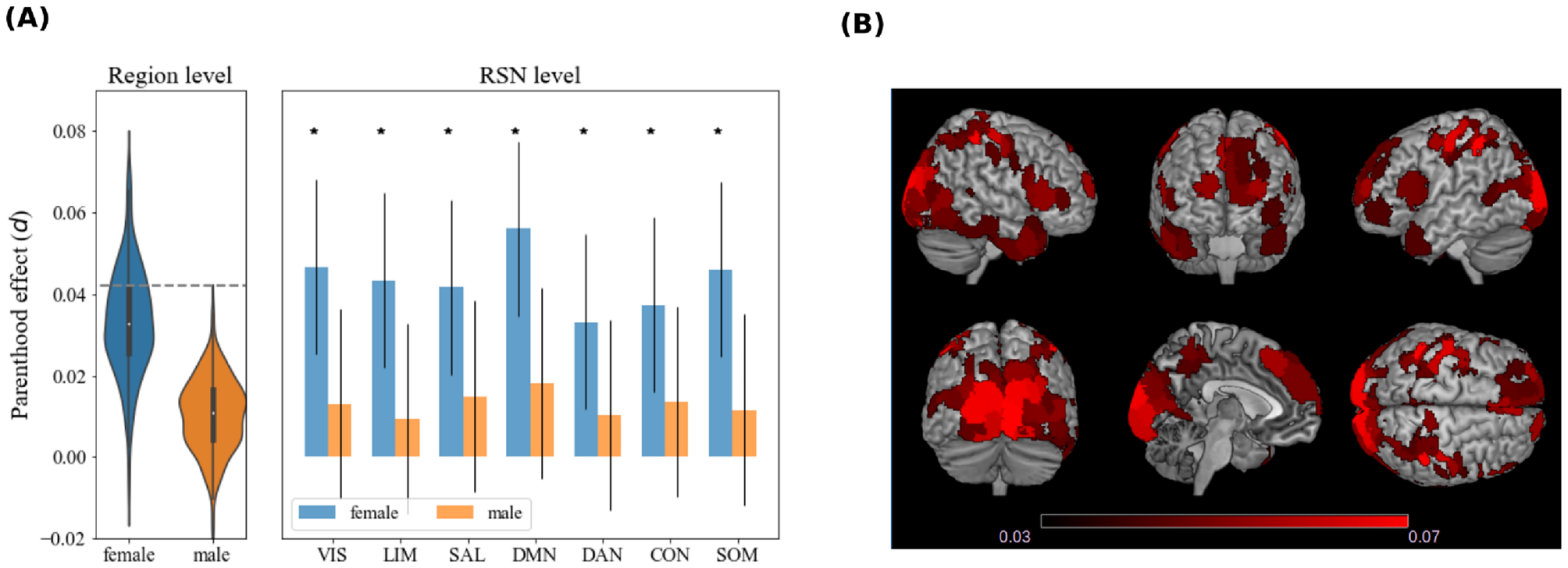
We considered 400 regions from the Schaefer atlas, and we performed a region-wise linear regression on grey matter density, separately for female and male participants. For each region, we tested the significance of the regressor associated with parenthood (t-test). We then grouped regions in seven resting-state networks (RSNs), and performed an RSN-wise linear regression (considering the mean grey matter density of all voxels belonging to regions of the same RSN), testing the significance of the regressor associated with parenthood (t-test). **(A)** Left: distribution of regional effect sizes (Cohen’s *d*) for males and females over the 400 regions. The dashed line represents the threshold for significance (t-test, $P<0.05$ corrected for 400 multiple comparisons). Right: RSN-wise effect sizes. Asterisks ($*$) mark significant effects (t-test, $P<0.05$ corrected for seven multiple comparisons). VIS: visual network; LIM: limbic network; SAL: salience network; DMN: default mode network; DAN: dorsal attention network; CON: control network; SOM: somatomotor network. **(B)** Brain rendering of the regions showing a significant effect for female participants.

We conducted a similar analysis on all 400 parcels defined in the Schaefer atlas^37^ to explore whether the motherhood effect was specific to the analyzed ROIs. Linear models (including all regressors) were globally significant: for all parcels, and for both males and females, we obtained $p < 11^{-11}$ (F-test, corrected for 400 multiple comparisons with Bonferroni). Among females, we identified a significant effect of parenthood on gray matter density in 99 of the 400 regions (t-test, $p<0.05$, corrected for 400 multiple comparisons). The observed significant effects (Cohen’s *d*) ranged from 0.042 to 0.072 (Fig. 2A). Globally over all ROIs, we identified an effect of parenthood (Cohen’s $d=0.046$, $p=2\cdot 10^{-5}$). For males, we did not observe any significant effect. Non-significant effects were mostly positive, with a maximum effect size of 0.037. Globally over all ROIs, we did not find a significant effect of parenthood on gray matter (Cohen’s $d=0.014$, $p=0.24$). The strongest effects for women were observed in the primary visual and motor cortex; other regions include the anterior and posterior cingulate cortex, the ventrolateral prefrontal cortex, and the temporal pole (Fig. 2B). We investigated whether the effect found affects primarily one or few resting state networks (RSNs). We repeated the analysis by aggregating regions belonging to each of the seven resting-state networks (RSNs) described by^37,38^. Effects are comparable across RSNs (Fig. 2A). We further examined the relationship between gray matter density and age. We observed a significant negative relationship between age and gray matter in 395 out of 400 regions. The age effect is much stronger than the parenthood effect: Cohen’s *d* for age is in the range $[-0.6,-0.1]$ as compared to [0.042, 0.072] for parenthood (Fig. S2 in the Supplementary Materials). Such a large magnitude justifies a-posteriori the care we took to control for age effects. Age effects exhibit a negative correlation with the parenthood effect (Pearsons’s r=-0.38). This suggests that brain regions undergoing a more significant age-related decline in grey matter exhibit a slower rate of decline in individuals who are mothers.

Next, we examined the potential influence of parity (number of children) alongside parenthood (presence/absence of a child). We repeated the analysis by substituting the dichotomic parenthood regressor with the parity regressor. The regressor includes 0, i.e., childless women. In our sample, parity was distributed as follows: 0 (22%), 1 (12%), 2 (44%), 3 (16%), 4 (4%), 5 (1%), $>5$ (1%). The parity effect was significant in 136 regions out of 400 (t-test, $p<0.05$ corrected for 400 multiple comparisons; Fig. S3a in the Supplementary Materials. The effects of parenthood and parity were highly correlated (Pearson’s $r=0.86$; Fig. S3b in the Supplementary Materials) across brain regions. When partialing out the effect of parenthood, the relationship between parity and gray matter was found to be non-significant across all 400 regions. This indicates that parenthood (i.e., the transition from parity $=0$ to parity $>0$) is the primary driver of the observed parity effect rather than the number of children. To check whether this is the case, we repeated the parity analysis by focusing on parents only (parity $\ge 1$) and using parity as a regressor. We find a significant effect of parity and grey matter for females (but not males) in 21 out of 400 regions (t-test, $P<0.05$ corrected for 400 multiple comparisons; Fig. S3c in the Supplementary Materials). The effect was evenly distributed across resting state networks (Fig. S3c in the Supplementary Materials), and it correlated with the effect of parenthood computed on the sample of parents and non-parents ($r=0.56$; Fig. S3d in the Supplementary Materials).

We further investigated whether the association between pregnancy and grey matter volume modifies with time elapsed since pregnancy. To remove possible confounds related to more than one pregnancy, we only considered the subset of women with only one child (i.e., parity = 1). We computed the time elapsed since first birth and tested for possible effects on grey matter. We used two time regressors for accounting for potential non-linear effects: one related to the time elapsed since first birth and one to its square. We found no significant effects between time elapsed since first birth (or its square) and grey matter (Fig. S4a in the Supplementary Materials), both when analyzing the 400 regions independently or aggregating them in resting-state networks. Analogously, we tested whether the age at first birth was associated with gray matter. We used two regressors, one related to the age at first birth and one to its square. We found no significant effects (Fig. S4b in the Supplementary Materials).

Finally, we tested for a possible association between pregnancy alone and grey matter. To this aim, we compared childless women who never had a pregnancy ($N=3485$) with childless women who suffered at least one episode of stillbirth, termination, or miscarriage ($N=872$). To control the possible confounding effects of age, we age-matched the two groups (similarly to what was done for parents/non-parents). The association between pregnancy alone and gray matter volume was insignificant in any of the 400 regions (Fig. S5a in the Supplementary Materials). The (non-significant) association between pregnancy and gray matter volume was weak ($d < 0.02$) and poorly correlated with those of parenthood (Pearson’s $r=0.06$; Fig. S5b in the Supplementary Materials).

### Parenthood and well-being

We employed a Probit model to examine the association between parenthood and well-being, incorporating marginal effects and robust standard errors to address heteroskedasticity. Consistent with existing literature^39^, well-being can be conceptually divided into a cognitive component and an affective component, encompassing both positive and negative affect. Our models comprehensively integrate these components to capture the relationship between motherhood and well-being. Specifically, we model four outcomes: (1) a binary measure representing the belief in the meaningfulness of one’s own life, reflecting the cognitive component of well-being; (2) a binary measure of self-assessed happiness, representing the positive affective component; and (3) two dummy variables with a value of 1 when respondents have experienced feelings of being fed-up or emotionally distressed in the past two weeks, capturing the negative affective components. While the measures of “fed-up” and “emotionally distresse” may seem less directly tied to child-rearing in older individuals, we included them to encompass, as suggested by the existing literature on the topic^40^, a comprehensive range of measures linked to emotional well-being, recognizing that parenthood’s effects can extend beyond immediate child-rearing responsibilities.

Table 2 encapsulates our primary findings after adjusting for 12 multiple hypotheses testing. Parenthood significantly enhances the sense of meaning for both sexs ($\beta = 0.060, p<0.001$ for women and $\beta = 0.101, p<0.001$ for men), highlighting the intrinsic value of child-rearing. The positive and significant interaction between male sex and parenthood further amplifies this sense ($\beta = 0.048, p<0.05$), suggesting a unique aspect of fatherhood. Moreover, marital status remains a robust predictor of well-being across dimensions, emphasizing the importance of supportive relationships. Control variables such as the number of people in the household and individual health status also significantly impact well-being, with larger household sizes and poorer health conditions associated with reduced well-being ($p<0.05$ to $p<0.001$).

Table 2 is complemented by an extensive evaluation through robustness checks detailed in Table S1 and S2 in the Supplementary Materials.

**Table 2:** Parenthood and wellbeing.

	(1)	(2)	(3)	(4)	(5)	(6)	(7)	(8)	(9)	(10)	(11)	(12)
	Happy			Sense of Meaning			Fed-up			Emotional Distress		
	Women	Men	Overall	Women	Men	Overall	Women	Men	Overall	Women	Men	Overall
Has at least one child (d)	0.001	0.031	− 0.001	0.060***	0.101***	0.056***	0.021	0.008	0.024	0.010	− 0.018	0.009
	(0.011)	(0.014)	(0.010)	(0.011)	(0.013)	(0.010)	(0.009)	(0.010)	(0.008)	(0.010)	(0.011)	(0.009)
Male			− 0.025			− 0.036			− 0.035*			− 0.137***
			(0.014)			(0.014)			(0.011)			(0.012)
Has at least one child=1 $\times$ Male			0.033			0.048*			− 0.021			− 0.029
			(0.015)			(0.016)			(0.012)			(0.013)
Married or in a partnership (d)	0.175***	0.178***	0.168***	0.068***	0.103***	0.081***	− 0.006	− 0.027	− 0.015	0.046**	− 0.030	0.016
	(0.014)	(0.018)	(0.010)	(0.014)	(0.018)	(0.010)	(0.011)	(0.013)	(0.008)	(0.012)	(0.014)	(0.009)
Number in household	− 0.023*	− 0.031**	− 0.026***	0.009	− 0.001	0.004	− 0.002	0.003	0.001	− 0.026***	0.004	− 0.013*
	(0.007)	(0.008)	(0.005)	(0.007)	(0.008)	(0.005)	(0.006)	(0.006)	(0.004)	(0.006)	(0.007)	(0.004)
Age	− 0.002	0.073***	0.007	− 0.009	0.057**	0.002	− 0.036***	− 0.050***	− 0.035***	0.008	0.023	0.009
	(0.010)	(0.016)	(0.007)	(0.009)	(0.015)	(0.007)	(0.007)	(0.011)	(0.006)	(0.008)	(0.012)	(0.006)
Age sq.	0.000	− 0.000***	− 0.000	0.000	− 0.000**	0.000	0.000***	0.000**	0.000***	− 0.000	− 0.000	− 0.000
	(0.000)	(0.000)	(0.000)	(0.000)	(0.000)	(0.000)	(0.000)	(0.000)	(0.000)	(0.000)	(0.000)	(0.000)
Health: Poor (d)	− 0.456***	− 0.474***	− 0.486***	− 0.469***	− 0.430***	− 0.437***	0.497***	0.512***	0.492***	0.260***	0.204***	0.244***
	(0.021)	(0.026)	(0.023)	(0.028)	(0.036)	(0.025)	(0.021)	(0.026)	(0.019)	(0.022)	(0.031)	(0.020)
Health: Fair (d)	− 0.402***	− 0.371***	− 0.379***	− 0.324***	− 0.311***	− 0.296***	0.343***	0.279***	0.289***	0.194***	0.135***	0.169***
	(0.013)	(0.016)	(0.010)	(0.015)	(0.018)	(0.010)	(0.013)	(0.015)	(0.008)	(0.011)	(0.014)	(0.009)
Health: Good (d)	− 0.178***	− 0.153***	− 0.155***	− 0.116***	− 0.094***	− 0.097***	0.125***	0.092***	0.100***	0.100***	0.046***	0.076***
	(0.011)	(0.013)	(0.007)	(0.010)	(0.012)	(0.007)	(0.009)	(0.009)	(0.006)	(0.009)	(0.010)	(0.007)
Townsend deprivation index	− 0.005	− 0.007**	− 0.006***	− 0.003	− 0.001	− 0.002	0.002	0.004*	0.003**	− 0.004	− 0.001	− 0.002
	(0.002)	(0.002)	(0.001)	(0.002)	(0.002)	(0.001)	(0.001)	(0.001)	(0.001)	(0.001)	(0.002)	(0.001)
N	13436	10233	23669	13307	10023	23330	18965	15265	34230	18982	15279	34261

The table reports coefficients and their standard errors. robust to heteroskedasticity. Columns 1–2, 4–5, 7–8, 10 – 11: Probit, marginal effects. Columns 3, 6, 9: OLS.* p*-values corrected for 12 multiple hypotheses testing. * p < 0.05, ** p < 0.01, *** p < 0.001

### Motherhood, the brain, and well-being

Building on our initial findings, which revealed no observable changes in fathers’ brains-both in the specific regions highlighted by^3^ and across the resting state networks (RSNs) previously examined—we narrowed our focus to investigate the unique intersection of motherhood and well-being. Our mediation model, designed to parse out the effects of motherhood on well-being into natural direct effects (NDE), natural indirect effects (NIE), and total effects (MTE), aimed to assess whether alterations in grey matter volumes within these regions serve as mediators in the relationship between motherhood and our four key well-being dimensions: happiness, sense of meaning, feelings of being fed-up, and emotional distress (Table 3). The analysis indicated no significant influence of motherhood on happiness and fed-upness through the examined brain networks, as evidenced by nonsignificant NDE, NIE, and MTE values. Conversely, a significant total effect (MTE) across all brain networks was found for the sense of meaning, confirming our previous results and highlighting a robust positive association with motherhood for this particular outcome. The absence of significant indirect effects (NIE) in our study indicates that alterations in grey matter volume within the examined regions likely do not constitute the principal mechanism by which motherhood enhances the sense of meaning. Conversely, in our investigation into emotional distress, we noted a negative indirect effect (NIE) within the default network and across the regions highlighted by^3^-areas that resonate with our initial theoretical framework. Regarding the Default network and the average regions identified by^3^, after adjusting for 32 multiple comparisons with Anderson’s correction^41^, the refined q-values were determined to be 0.06 and 0.08, respectively. In comparison, the Bonferroni corrected *p*-values were calculated to be 0.08 and 0.09. These adjustments were made from the original, uncorrected *p*-values of 0.0025 and 0.0031. While this result does not provide evidence for the presence of an effect, it does not provide strong evidence for its absence either, thereby leaving the specific question undecided. Future studies on independent data shall clarify the issue.

**Table 3:** Mediation analysis results.

	Effect	Happy	Sense of meaning	Fed-up	Emotional distress
Average	NDE	0.001	0.057**	0.021	0.012
	NIE	0.000	0.000	0.000	− 0.001
	MTE	0.001	0.058**	0.021	0.011
Dorsal Attention	NDE	0.001	0.058**	0.020	0.011
	NIE	0.000	0.000	0.000	0.000
	MTE	0.001	0.058**	0.020	0.011
Control	NDE	0.001	0.058**	0.021	0.011
	NIE	0.000	0.000**	0.000	0.000
	MTE	0.001	0.058**	0.020	0.011
Default	NDE	0.001	0.057**	0.021	0.012
	NIE	0.000	0.001	0.000	− 0.001
	MTE	0.001	0.058**	0.021	0.011
Visual	NDE	0.001	0.057**	0.021	0.012
	NIE	0.000	0.000	0.000	− 0.001
	MTE	0.000	0.057**	0.021	0.011
Limbic	NDE	0.001	0.058**	0.020	0.011
	NIE	− 0.001	0.000	0.000	− 0.001
	MTE	0.001	0.058**	0.020	0.010
Salience	NDE	0.000	0.058**	0.021	0.011
	NIE	0.000	0.000	0.000	0.000
	MTE	0.000	0.058**	0.021	0.011
Somatomotor	NDE	0.001	0.058**	0.021	0.011
	NIE	0.000	0.000	0.000	0.000
	MTE	0.000	0.058**	0.021	0.011

The table reports the coefficients of the mediation analysis. Significance levels have been adjusted for multiple comparisons (32 tests). * $${{p}}<0.05$$, ** $${{p}}< 0.01$$, *** $${{p}}< {0.001^{**}}$$. NDE (Natural Direct Effect) represents the influence of motherhood on well-being without the mediation of brain networks. NIE (Natural Indirect Effect) indicates the association between motherhood and well-being through the mediator. MTE (Total Effect) represents the sum of direct and indirect effects. Results are not standardized

## Discussion and conclusions

Motherhood is a primary transformative event not only for mothers’ lives but also for mothers’ brains. Longitudinal studies have consistently demonstrated a reduced gray matter among new mothers, particularly in regions associated with social cognition. These modifications are interpreted as adaptive responses, facilitating cognitive and affective processing and promoting attachment to the newborn^3^. The brain changes have been documented in a small group of mothers persisting up to six years post-birth^23^.

Our cross-sectional study explored the long-term impact of motherhood on the brain, revealing discernible differences between mothers and non-mothers even five decades after giving birth (see Fig. S4 in the Supplementary Materials). However, the sign of the difference was the opposite of the one reported by longitudinal studies^3,23^. We detected an *increase* of gray matter density where a *decrease* was shown previously. Furthermore, we showed that the increase in gray matter density is not limited to a few brain regions or a single network, but instead, it is widely distributed across cortical and subcortical structures, even though it is particularly strong in the occipital and frontal lobes. The positive association between pregnancy and gray matter density persisted even after considering multiple socio-economic variables that may also have a “neuro-protective” role.

Our findings support the hypothesis of a U-shaped pattern of gray matter modifications throughout mothers’ reproductive life: an initial decline is followed by a subsequent increase that surpasses the early reduction^18,42^. This trajectory suggests the influence of multiple, temporally distinct forces shaping the maternal brain. During pregnancy and puerperium, rapid brain modulation targets brain regions crucial for maternal care, possibly through synaptic pruning and the associated gray matter reduction. Later, motherhood exerts a broader protective effect against the physiological gray matter loss due to aging, hence the observed gray matter increase compared to similar-age childless women. This effect might be attributed to the fact that parents, on average, experience an enriched cognitive and relational environment associated with parenthood, and are less likely to be exposed to the detrimental impact of social isolation^43^. Our findings are consistent and further specify previous reports showing that motherhood keeps mothers’ brains younger^5,7,11,12^.

Our study has not fully captured the U-shaped gray matter density pattern in women likely because of the extended time elapsed since most participants’ last pregnancy. In our sample, 86% of women were more than 20 years postpartum. Consequently, the initial decrease in gray matter density likely occurred outside the observable window of our study. Therefore, our findings reflect the later portion of the U-shaped trajectory, where gray matter volume stabilizes after an increase. Future research aiming to characterize the response dynamics comprehensively should consider including participants at various stages following childbirth (or employ longitudinal designs with longer follow-up periods). Specifically, recruiting participants closer to the childbearing period could provide valuable insights into the maternal brain changes over time.

The experience of parenthood and being exposed to an enriched cognitive and relational environment can protect against age-related gray matter loss, we would expect to see this effect in fathers as well. However, our analysis of the UK BioBank dataset did not show a significant association between fatherhood and gray matter density. There could be multiple reasons for this finding.

One possible explanation is that the effects of early and late parenthood may have a biological basis. Pregnancy triggers a series of sex-specific changes that initially lead to a reduction in gray matter. However, these changes may have a broader protective effect in the long run. The initial sex-specific trigger may also influence the lasting impact of these changes.

An alternative explanation is that fathers may not entirely reap the benefits of an enriched environment due to their historically lower involvement in childcare^44–46^. Should this be the case, a more equitable distribution of long-term protective effects across sexes will emerge once this behavioral imbalance disappears. Indeed, recent studies have shown that fathers in more contemporary cohorts are experiencing the effects of parenthood, supporting this hypothesis^21,22^. Future research should scrutinize the allocation of caregiving responsibilities between mothers and fathers and its influence on neurobiological adaptations.

A third explanation could be that the influence of fatherhood may exist but remains undetected through gray matter density metrics. Research utilizing UKBiobank data revealed a neuroprotective effect among parents, encompassing both mothers and fathers^12^. The divergence in findings between our study and that of^12^ could stem from at least three methodological variances. First, we assessed gray matter density across distinct subregions independently, as opposed to^12^ approach, which employed a composite measure (including over 403 brain features) for predicting brain age. Second, the two samples are not fully overlapping, due to the selection of the variables and inclusion criteria. Third, the selection of covariates differed between the two studies. Intriguingly, whereas^12^ observed no variance in brain aging between mothers and fathers, our findings suggest otherwise, pointing to potential sex-specific factors influencing brain age predictions. Future research is warranted to explore these sex-related differences further.

To further investigate the two-phase model of the brain response to motherhood, where the modified family environment potentially influences the later phase, we hypothesized that women who experienced stillbirth, termination, or miscarriage would not exhibit the “motherhood effect” on the brain structure. This group, lacking the ongoing caregiving experience, could help isolate the specific effects of pregnancy on the brain. Our findings support this hypothesis, demonstrating no significant differences in brain structure between this group and nulliparous controls. However, it is important to acknowledge the limited sample size of this specific group and the high diversity of the physiological and emotional impacts of different pregnancy outcomes. These factors could have potentially determined the negative finding and necessitate a cautious interpretation of our results.

Our study also documented possible consequences of parenthood on well-being. Specifically, we observed a positive correlation between parenthood and cognitive well-being, as indicated by a stronger belief that one’s life is meaningful. Conversely, we found no significant correlation between parenthood and negative affect. While few studies have focused on studying parental well-being in older ages, our results align with those reported in^40^ which found, in a sample of men and women at midlife to old age from Norway, that parental status has an effect on women’s cognitive well-being (life satisfaction and self-esteem) but not on affective wellbeing (positive and negative affect, depression, loneliness).

Finally, we explored whether the modifications in mothers’ brains could have a role in producing the observed effects on mothers’ well-being. To this aim, we run a mediation analysis. Given the cross-sectional nature of our data, finding a mediation role of brain changes over well-being would not prove a causal relationship between the two but, more weakly, would be consistent with it. Conversely, a negative finding would provide evidence for the absence of a causal relation. As in the previous analyses we considered the brain regions identified in the study by^3^, and the resting state networks (RSNs) that exhibited differences between mothers and non-mothers in our analysis. The mediation analysis confirmed the absence of motherhood effects on happiness and feelings of being fed-up and the association between motherhood and sense of meaning shown by the Probit analysis. Furthermore, and importantly, the analysis excluded a mediation role of the gray matter changes on the sense of meaning, suggesting that the changes in grey matter volume are not the primary mechanism through which motherhood elevates the sense of meaning. Other measures and causal paths should be explored to explain that outcome.

In summary, our study findings are consistent with a distinctive U-shaped trajectory of gray matter density in mothers’ brains, indicating an initial decline in specific brain regions followed by a subsequent wide-range increase due to protective effects against age-related gray matter loss. Fathers did not exhibit this pattern, which may be attributed to the sex-specific biological underpinnings of these effects, to the relatively lower caregiving involvement in the cohort studied, or to the importance of other physiological measures to detect the effect. Psychologically, motherhood is associated with an increased sense of meaning. Structural brain alterations alone do not solely determine nor mediate the impact of motherhood on well-being, prompting the need to explore other potential causal pathways. Acknowledging this, future research should integrate additional mediators, offering a more comprehensive exploration of how motherhood influences emotional well-being. These mediators encompass aspects not accounted for in our study due to data constraints, such as social support, coping strategies, and cultural norms. Broadening the analytical scope to encompass these dimensions will facilitate a more thorough understanding of the intricate interplay between motherhood, brain modifications, and emotional well-being, ultimately providing deeper insights into the complex links between motherhood and well-being.

## Methods

### Motherhood and the brain

We rely on data from the UK Biobank, a rich, long-term prospective epidemiological study of 500,000 volunteers aged 45–82 at MRI scanning (Fig. S1 in the Supplementary Materials). On the suitability of this data source, see^5^ and^7^. The UK Biobank includes genome-wide genetic data using a purpose-designed genotyping array and brain scans for around 100,000 participants. The data include image-derived phenotypes (IDPs), and among those, the volume of grey matter in distinct brain regions and measures of functional and structural connectivity between specific pairs of brain areas. Furthermore, the UK Biobank includes survey questions on cognitive and affective well-being. We removed from the samples participants who did not participate in the MRI acquisition, participants with known brain disorders, including dementia, and participants with mild cognitive disorder, which includes cognitive decline involving learning, memory, or concentration. Finally, we excluded subjects for which values of one or more regressors were unavailable (see below). These criteria restricted our sample to $n=36655$ (19043 females; 17612 males).

Procedures for brain imaging acquisition and initial quality check have been described in^47,48^, and further details are given in the UK Biobank Brain Imaging documentation^49^. T1-weighted structural images were acquired through a standard Siemens Skyra 3T, with a standard Siemens 32-channel RF receive head coil. A 3D MPRAGE, sagittal, in-plane acceleration iPAT = 2, prescan-normalized imaging protocol was used, with a total duration of 5 minutes and voxel resolution of 1 × 1 × 1 mm. Upon dicom to nifti conversion and defacing, preprocessing steps included: coregistration and gradient distortion correction using BET and FLirt; nonlinear warping to MNI152 space using FNIRT; brain extraction through a standard mask; tissue segmentation and bias field correction using FAST.

*ROI analysis*. We considered the group significance map shown in ^3^, Fig. 1, and publicly available at https://figshare.com/articles/dataset/Source_files_figures_Hoekzema_et_al_/4216809. Following Hoekzema et al., the map was thresholded at $T=5.4029$, corresponding to $p < 0.05$, family-wise error (FWE)-corrected. This resulted in 11 separated clusters, with size and location as given by ^3^, Table 1. The cluster map (originally in 0.5 mm $\times$ 0.5 mm $\times$ 0.5 mm MNI space) was resampled at 2 mm $\times$ 2 mm $\times$ 2 mm for consistency with (masked, normalized, smoothed) T1 maps. For each participant, we estimated the average gray matter volume in the 11 clusters by overlapping the cluster map with the structural image and averaging the image values for all voxels inside the cluster.

*Age matching*. Of 19043 female participants, 4194 were non-parents (parity = 0) and 14849 were parents (parity $> 0$). To age-match female parents and non-parents, we subsampled the group of parents such that the age distribution approximately matched that of non-parents. We grouped ages in 7 discrete categories ($\le$ 50, 51–55, 56–60, 61–65, 66–70, 71–75, $\ge 76$). Each non-parent participant was randomly matched to a parent participant within the same age category, which yielded an age-matched subsample of 4194 parents. The same procedure was repeated for male participants (n=17612), obtaining two age-matched samples of 3494 male parents and non-parents.

*Regression*. The regressors in the analysis included the variable “parenthood”, coded as 0 for childless individuals and 1 for parents. Additionally, several nuisance variables were included, namely age, the square of age, Townsend deprivation index (a composite measure used to assess socioeconomic disadvantage within specific geographical areas, incorporating indicators such as unemployment, non-home ownership, household overcrowding, and low social class ), general health status, and household size. Despite the matching of participants based on age, including age and its square in the regression model was still necessary. This was done to account for potential linear and non-linear relationships between age and other regressors, such as household size, ensuring that age-related factors were properly controlled. Following common practice ^50^, we tested for significance of each regressor’s association using a *t*-test (if *N* is the number of observations, *p* the number of regressors, $\beta$ the regressor’s estimate, and $\sigma _\beta$ its error, the *T* variable is computed as $T=\frac{\beta \sqrt{N-p}}{\sigma _\beta }$). The effect size is measured using Cohen’s *d*, computed as $d=T/\sqrt{N-p}=\frac{\beta }{\sigma _\beta }$.

### Motherhood and well-being

We estimate the association between parenthood and happiness by sex by estimating an equation similar to (1) in which the dependent variables are different measures of well-being, as suggested in^40^.

1 $$\begin{aligned} SWB_{i}=\beta _0+\beta _1 HasChild_{i}+X_{i}+\varepsilon _{i} \end{aligned}$$

These measures include the belief that own life is meaningful measured on a 5-item Likert scale (ranging from not at all to an extreme amount), which has been recorded as a binary variable assuming value when one the respondent believes that their life is meaningful at least to a moderate amount; a self-assessed measure of happiness measured on a 6-items Likert scale (ranging from extremely unhappy to extremely happy) recoded as a binary variable assuming value 1 when the respondent is at least moderately happy; and a dummy variable taking value 1 when the respondent has felt to be fed-up or has experienced emotional distress in the last two weeks (“Over the last 2 weeks, how often have you been bothered by any of the following problems? fed-up feeling”). As for this second part of the analysis, the set *X* of covariates includes the above-mentioned covariates. In robustness models (Table S1 and S2) we also include a set of lifestyles^51^, media-consumption^52^, and relationships-related variables^53^ which are related to well-being in previous studies: Average hours slept, alcohol intake frequency, average hours spent outdoors, average hours spent watching TV, average hours spent driving, the number of days per week of vigorous physical activity (10+ minutes), and the frequency of friend/family visits. We ensure the reliability of our estimates by using robust standard errors, making our results resilient to heteroskedasticity. Standard errors are robust to heteroskedasticity.

**Figure 3:**
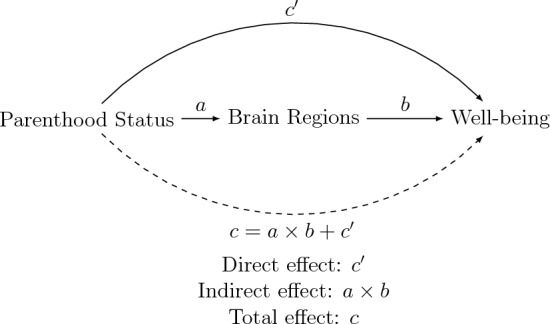
Mediation graph.

In our investigation, we deploy a mediation analysis framework to dissect the intricate processes by which parenthood could influence well-being, visually illustrated in the mediation graph within Figure 3. This framework distinguishes between the direct and indirect effects of parenthood on well-being. The direct effect symbolized as $c'$, delineates the pathway from parenthood status directly to the well-being indicator, excluding any intermediary brain regions. It quantifies the association between parenthood and well-being, devoid of the mediating influence of brain changes. In contrast, the indirect effect unfolds through two sequential paths: the pathway from parenthood status to brain regions (denoted as $a$) and from brain regions to the well-being indicator (denoted as $b$). The product of these pathways ($a \times b$) computes the mediation effect, elucidating the proportion of the effect of parenthood on well-being that is channeled through the changes in brain regions. The total effect, represented as $c$, aggregates the influences of both the direct pathway from parenthood to well-being and the mediated pathway through brain regions, visually underscored by a dashed arrow. Importantly, the absence of a statistically significant direct link ($c'$) between the independent variable (parenthood) and the dependent variable (well-being) does not negate the presence of a meaningful indirect effect through the mediator. Sometimes, the entirety of an independent variable’s effect on an outcome is mediated. Grasping the mechanisms underlying an association is a principal aim of mediation analysis. The discovery of a significant indirect pathway, even without a direct association, can shed light on the intricate processes linking the independent variable to the outcome. Conversely, conducting a mediation analysis when there is no detectable association between the independent variable and the mediator (a) is not advisable. For example, if one hypothesizes a causal path starting from motherhood that causes brain changes that, in turn, cause behavioral/emotional changes, then one should expect to observe both a significant total effect $c$ and a significant mediation effect $c'$. The failure to observe such an effect would weaken the above-mentioned hypothesis.

Heeding the insights of^54^, we recognize that even in randomized studies, identifying average treatment effects (ATE) does not automatically elucidate the nature of direct and indirect effects without invoking further assumptions. Given the cross-sectional design of our dataset and the impracticality of randomly assigning individuals to parenthood, our analysis is subject to potential biases from unobserved heterogeneity and reverse causality. For instance, individuals predisposed towards happiness may be more likely to embark on parenthood^55^, suggesting that our findings are best understood as associations rather than causative linkages.

To assess the mediation effect within these constraints, we employ the methodological framework developed by^56^, which extends the seminal contributions of^57^ and^58^. Our application of mediation analysis utilizes parametric regression models facilitated by the paramedics command in Stata 17^59^.

### Supplementary Information

Supplementary Information.

## Data Availability

Access to the UK Biobank can be obtained by submitting an application, with further details provided at https://www.ukbiobank.ac.uk. Following our commitment to transparency and reproducibility, we will make the associated code available on the Open Science Framework Platform upon acceptance of the manuscript.
