# Supplementary material for: Enduring maternal brain changes and their role in mediating motherhood’s impact on well-being
Source: Sci Rep. 2024 Jul 18;14:16608. doi: 10.1038/s41598-024-67316-y (PMC11258333; doi:10.1038/s41598-024-67316-y)
Supplement: Supplementary file 1 — Supplementary Information. [file 41598_2024_67316_MOESM1_ESM.pdf]

Supplementary Materials

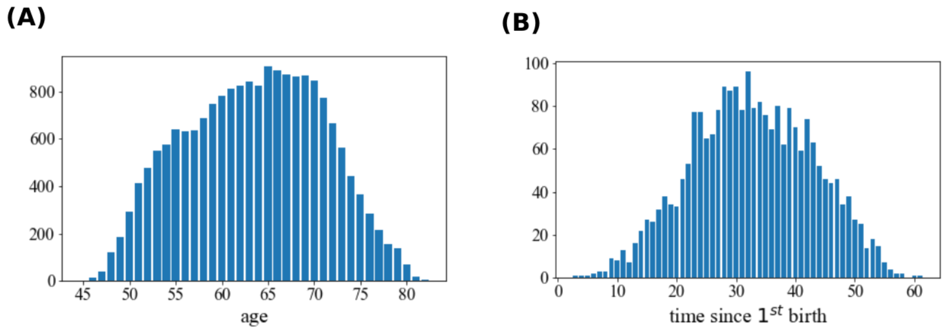

Figure S1: **(A)** Distribution of age in our sample of female participants **(B)** Distribution of time passed since the birth of the first child (years) in our sample of mothers

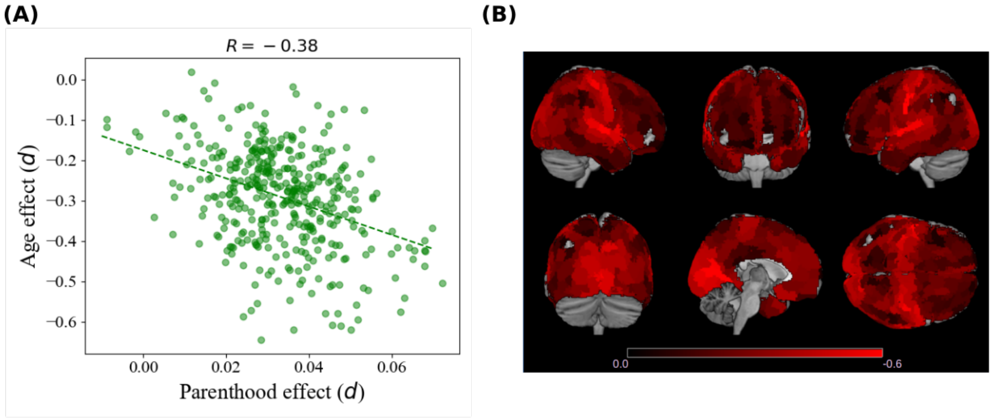

Figure S2: **(A)** Scatter plot illustrating variations in the effects of age and parenthood (Cohen's d) observed across the 400 regions of the Schaefer atlas for female participants. **(B)** Brain rendering highlighting regions that exhibit a significant association with age in female participants.

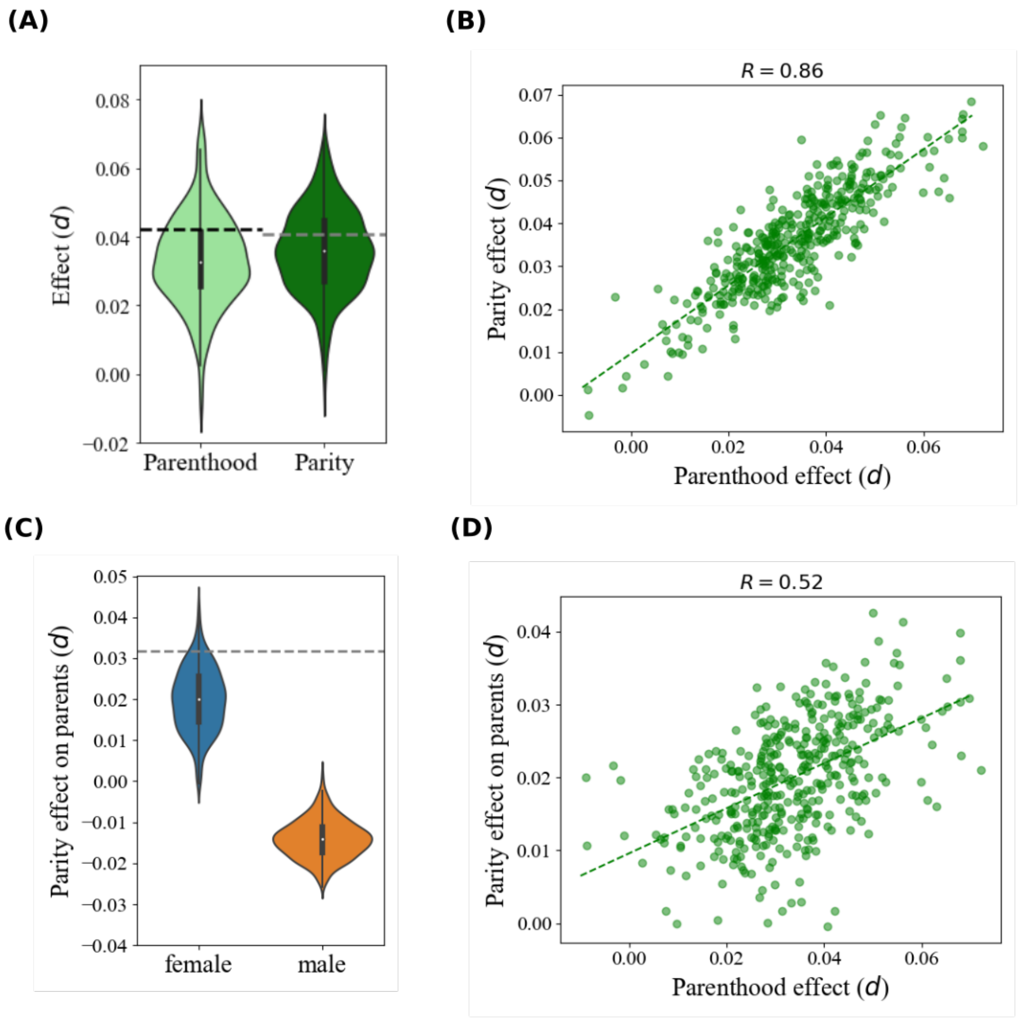

Figure S3: Association between parity and gray matter, examined with both parents and age-matched non-parents (A and B), and exclusively with parents (C and D). **(A)** Distribution of regional effect sizes (Cohen's d) for parenthood and parity across the 400 regions of the Schaefer atlas. Parity analysis includes parents (parity ≥ 1) and non-parents (parity $= 0$). Dashed lines indicate significance thresholds (t-test, $P < 0.05$ corrected for 400 multiple comparisons). **(B)** Scatter plot illustrating the relationship between parenthood and parity effects (Cohen's d) observed across the 400 regions of the Schaefer atlas for female participants. **(C)** Distribution of regional effect sizes of parity (sample including parents only) for males and females across the 400 regions of the Schaefer atlas. The dashed line represents the significance threshold (t-test, $P < 0.05$ corrected for 400 multiple comparisons). **(D)** Scatter plot illustrating the relationship between parenthood (sample including parents and non-parents) and parity effects (sample including parents only) observed across the 400 regions of the Schaefer atlas for female participants. Effects are reported using Cohen's d.

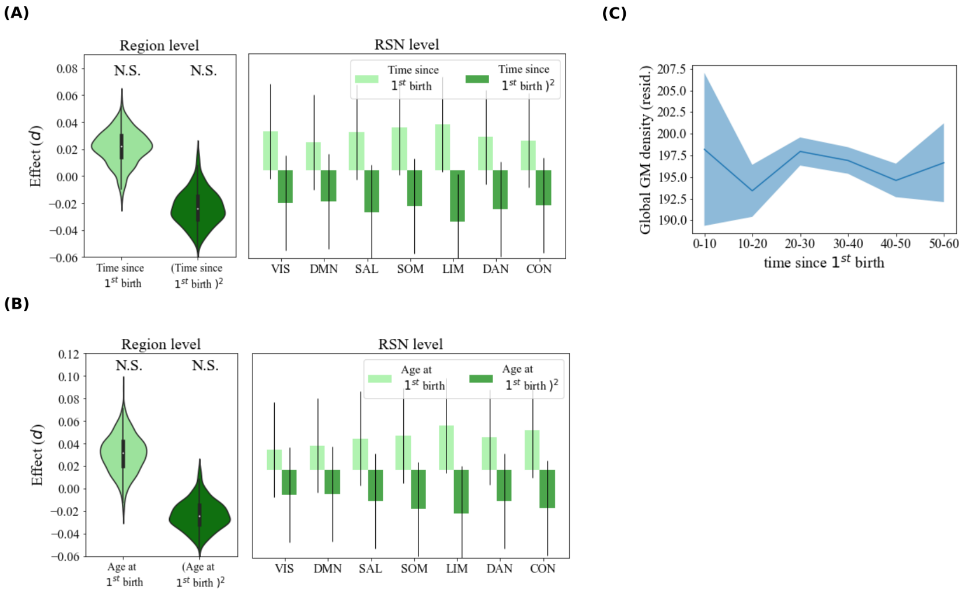

Figure S4: **(A)** Left: Distribution of regional effect sizes (Cohen's d) for time elapsed since first birth and its square for the 400 regions of the Schaefer atlas. Right: effect sizes (Cohen's d) for time elapsed since first birth and its square for the seven resting state networks (RSN) considered in the study. **(B)** Left: Distribution of regional effect sizes (Cohen's d) for the age at first birth and its square for the 400 regions of the Schaefer atlas. Right: effect sizes (Cohen's d) for the age at first birth and its square for the seven resting state networks (RSN). **(C)** Average global grey matter density as a function of time elapsed since first birth. We considered the residual after regressing all nuisance variables, including age and age^2).

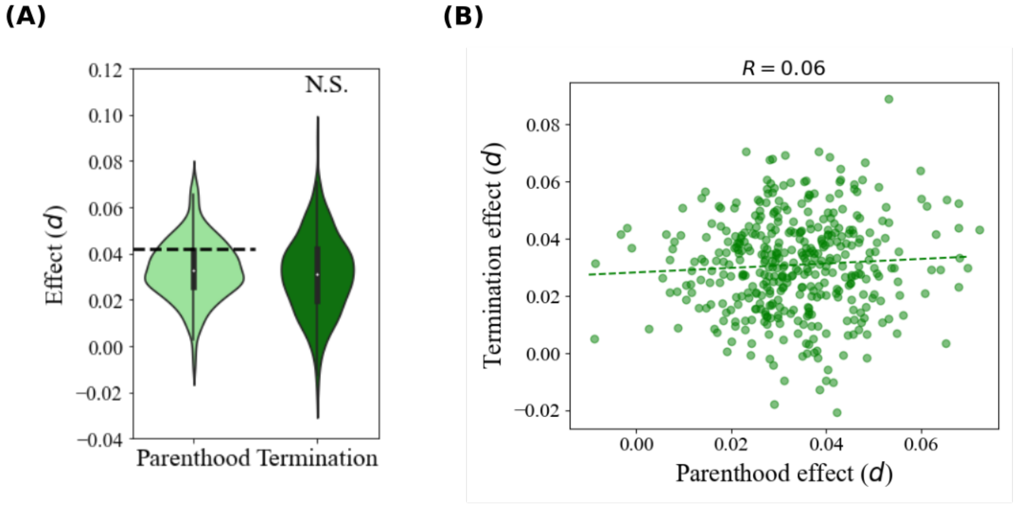

Figure S5: **(A)** Distribution of regional effect sizes (Cohen's d) for parenthood and termination(incomplete pregnancy) for the 400 regions of the Schaefer atlas. Dashed lines indicate the thresholds for significance (t-test, $P < 0.05$ corrected for 400 multiple comparisons). **(B)** We compare the effects (Cohen's d) of parenthood and aborted pregnancy across the 400 regions of the Schaefer atlas for female participants.

Table S1: Associations Between Motherhood and Well-being Measured as Happiness, Sense of Meaning, Feelings of Being Fed-up and Emotional Distress

	(1) Happy b/se	(2) Sense of Meaning b/se	(3) Fed-up b/se	(4) Emotional Distress b/se
		**Panel 1: Additional Controls**		
Has at least one child	0.001	0.063***	0.022*	0.001
	(0.011)	(0.011)	(0.009)	(0.009)
		**Panel 2: Age matched**		
Has at least one child	0.001	0.063***	0.022*	0.001
	(0.011)	(0.011)	(0.009)	(0.009)
		**Panel 3: Parity**		
Number of live births	0.003	0.024***	0.003	-0.000
	(0.004)	(0.004)	(0.003)	(0.003)
Observations	13432	13305	18965	18982
		**Panel 3: Parity (Parents only)**		
Number of live births	0.007	0.019**	-0.006	-0.007
	(0.006)	(0.006)	(0.005)	(0.005)
Observations	10339	10254	14801	14812

Note: Probit model, marginal effects. Robust standard error reported in brackets. * p<0.05, ** p<0.01, *** p<0.001 . Panel 1 integrates a comprehensive set of control variables—spanning lifestyle choices, media consumption habits, and relationship dynamics—known to influence well-being, as detailed in the Methods section. Panel 2 implements age-matching among mothers to ensure a more accurate comparison. Panel 3 delves into the effects of the number of children (parity), offering a nuanced view beyond mere parental status. Lastly, Panel 4 combines considerations of parity with parental status, providing a layered understanding of their collective impact on well-being.

Table S2: Associations Between Fatherhood and Well-being Measured as Happiness, Sense of Meaning, Feelings of Being Fed-up and Emotional Distress

	(1)	(2)	(3)	(4)
	Happy	Sense of Meaning	Fed-up	Emotional Distress
	b/se	b/se	b/se	b/se
	**Panel 1: Additional Controls**			
Has at least one child	0.012	0.078***	0.017	-0.007
	(0.014)	(0.014)	(0.010)	(0.011)
	**Panel 2: Age matched**			
Has at least one child	0.130*	0.465***	0.044	-0.078
	(0.058)	(0.059)	(0.053)	(0.047)
	**Panel 3: Parity**			
Number of live births	0.007	0.027***	-0.002	-0.005
	(0.005)	(0.004)	(0.003)	(0.004)
Observations	10233	10023	15265	15279
	**Panel 3: Parity (Parents only)**			
Number of live births	0.001	0.008	-0.008	-0.002
	(0.006)	(0.006)	(0.005)	(0.005)
Observations	8326	8173	12489	12500

Note: Probit model, marginal effects. Robust standard error reported in brackets. * p<0.05, ** p<0.01, *** p<0.001 . Panel 1 integrates a comprehensive set of control variables – lifestyle choices, media consumption habits, and relationship dynamics – known to influence well-being, as detailed in the Methods section. Panel 2 implements age-matching among mothers to ensure a more accurate comparison. Panel 3 delves into the effects of the number of children (parity), offering a nuanced view beyond mere parental status. Lastly, Panel 4 combines considerations of parity with parental status, providing a layered understanding of their collective impact on well-being.
